# A Comparative Genomic and Transcriptomic Survey Provides Novel Insights into *N*-Acetylserotonin Methyltransferase (ASMT) in Fish

**DOI:** 10.3390/molecules22101653

**Published:** 2017-10-02

**Authors:** Kai Zhang, Zhiqiang Ruan, Jia Li, Chao Bian, Xinxin You, Steven L. Coon, Qiong Shi

**Affiliations:** 1 BGI Education Center, University of Chinese Academy of Sciences, Shenzhen 518083, China; zhangkai@genomics.cn; 2 Shenzhen Key Lab of Marine Genomics, Guangdong Provincial Key Lab of Molecular Breeding in Marine Economic Animals, BGI Academy of Marine Sciences, BGI Marine, BGI, Shenzhen 518083, China; ruanzhiqiang@genomics.cn (Z.R.); lijia1@genomics.cn (J.L.); bianchao@genomics.cn (C.B.); youxinxin@genomics.cn (X.Y.); 3 BGI-Zhenjiang Institute of Hydrobiology, BGI Marine, Zhenjiang 212000, China; 4 Molecular Genomics Core, *Eunice Kennedy Shriver* National Institute of Child Health and Human Development, National Institutes of Health, Bethesda, MD 20892, USA; 5 Molecular Genomics Laboratory, National Institutes of Health, Bethesda, MD 20892, USA

**Keywords:** *N*-acetylserotonin methyltransferase, melatonin, phylogenetic analysis, comparative genomic and transcriptomic survey, fish

## Abstract

Melatonin is a multifunctional bioactive molecule that plays comprehensive physiological roles in all living organisms. *N*-acetylserotonin methyltransferase (ASMT, also known as hydroxyindole *O*-methyltransferase or HIOMT) is the final enzyme for biosynthesis of melatonin. Here, we performed a comparative genomic and transcriptomic survey to explore the *ASMT* family in fish. Two *ASMT* isotypes (*ASMT1* and *ASMT2*) and a new ASMT-like (*ASMTL*) are all extracted from teleost genomes on the basis of phylogenetic and synteny analyses. We confirmed that C-terminal of the ASMTL proteins (ASMTL-ASMT) is homology to the full length of ASMT1 and ASMT2. Our results also demonstrate that the two *ASMT* isotypes and their distribution in teleosts seem to be the result of combinations of whole-genome duplication (WGD) and gene loss. Differences were also observed in tissue distribution and relative transcript abundances of *ASMT1*, *ASMT2* and *ASMTL* through transcriptomic analysis. Protein sequence alignment and 3D structure prediction of ASMTs and ASMTL suggest differential roles for these *ASMT* genes. In summary, our current work provides novel insights into the *ASMT* genes in fish by combination of genomic and transcriptomic data.

## 1. Introduction

Melatonin is a multifunctional bioactive molecule that regulates circadian rhythms and seasonal reproductive processes; it is mainly synthesized rhythmically and secreted by the pineal gland and retinae [1]. This rhythmic secretory pattern, highly conserved in all vertebrates, is determined by the precise circadian regulation of melatonin biosynthesis [2]. Melatonin biosynthesis from the amino acid tryptophan (Trp) involves four enzyme-catalyzed reactions [3]. Firstly, Trp is transformed into 5-hydroxytryptophan (5-HTrp) by tryptophan hydroxylase (TPH, EC 1.14.16.4). Subsequently, 5-HTrp is converted to 5-hydroxytryptamine (serotonin) by the cytoplasmic enzyme dopa decarboxylase (DDC, also known as aromatic L-amino acid decarboxylase AAAD, EC 4.1.1.28). The third step is the transformation of serotonin by aralkylamine *N*-acetyltransferase (AANAT, EC 2.3.1.87) to *N*-acetylserotonin (NAS). The last step is completed by transforming NAS to melatonin (*N*-acetyl-5-methyoxytryptamine) with acetylserotonin *O*-methyltransferase (ASMT, also known as HIOMT, EC 2.1.1.4). As the final enzyme in the pathway of melatonin biosynthesis, ASMT is probably responsible for the seasonal variations in the melatonin secretion rhythm [4,5]. In addition, ASMT is considered a rate-limiting enzyme in melatonin synthesis since it provides an upper limit to the overall production rate once AANAT has been activated [6,7].

ASMT, identified in both animals and plants, belongs to the methyltransferase superfamily [8,9,10]. In plants, rice ASMT comprises three isotypes (*ASMT1*, *ASMT2*, and *ASMT3*) and all of the isotypes could encode ASMT activity, and overexpression of them could lead to the overproduction of melatonin in rice [8,11,12]. In mammals, the *ASMT* gene is located on the X chromosome [9,13]. The human *ASMT* gene possesses three possible isoforms, which have resulted from the alternative splicing of exons 6 and 7. Isoform 1 catalyzes the traditional transfer of a methyl group onto *N*-acetylserotonin, producing melatonin; however, isoforms 2 and 3 lack this enzyme activity [14]. In contrast to tetrapods, two *ASMT* genes have been reported in fish genomes, and may have been generated by the teleost-specific whole genome duplication (WGD) [15].

It was reported that the ASMT enzyme mainly exists in the retinae and pineal gland of the European sea bass [14]. ASMT2 was detected in several peripheral tissues, including liver and gut in teleost [10]. The existence of melatonin synthesis in gut and liver of goldfish has been demonstrated [15]. Recent studies suggested high expression of gut *ASMT* in zebrafish (*Danio rerio*) and rainbow trout (*Oncorhynchus mykiss*) [16,17]. Findings on a tropical carp (*Catla catla*) recently indicated that abundant *ASMT* mRNAs were detected in the gut and the transcription level of *ASMT* in brain displayed a significant negative correlation with water temperature [18]. Moreover, the involvement of brain melatonin in the modulation of seasonal reproductive parameters through the putative hypothalamo-pituitary-gonadal axis is suggested by high expression of *ASMT* in the brain during the preparatory phase [19,20,21].

However, a previous study [22] reported a new gene, acetylserotonin methytransferase-like (*ASMTL*) in animals, which is significantly homologous to the putative *ASMT* genes. In fact, *ASMTL* is the product of a gene fusion event, during which two different full-length genes (*maf* and *ASMT*) have been joined to form one single gene [22]. In contrast to those known *ASMT* genes, which are associated with the pathway of melatonin biosynthesis, the function of *ASMTL* is unclear as of yet. Interestingly, there are more clues to support the existence of *ASMTL* in sequenced fish genomes. For example, in the GenBank database, accession numbers NC_007120.7 (chromosome 9 of zebrafish) and NC_019879.1 (chromosome 21 of medaka) reveal the sequences for ASMTL.

Although two putative *ASMT* isotypes and *ASMTL* have been reported in fish, details of their evolution, 3D structures, tissue distribution and potential functions of these genes are still unknown. In addition, previous studies involved only a few bony fish, and no whole genome data were employed. With advances in next-generation sequencing capabilities, genomic and transcriptomic data of many fish species are now available, and with rapid developments in bioinformatics, this sequence information is more valuable. We can access fish *ASMT* genes (and their encoded protein sequences) from many published and unpublished whole genome sequences, including amphibious (mudskippers), cave-restricted or eyeless (*Sinocyclocheilus anshuiensis*), and tetraploid (*Sinocyclocheilus spp.*) fishes. This allows us to determine species-specific presence or absence of ASMT isotypes, as well as sequence differences across the species. In this report, we analyzed the protein sequence differences in fish *ASMT* isotypes and *ASMTL*, and constructed a phylogenetic tree with a synteny analysis. Furthermore, we also analyzed the exon-intron structures of these three genes, and obtained full-length *ASMT1*, *ASMT2,* and *ASMTL* in two representative mudskipper species by molecular cloning. Finally, transcriptome analysis was applied to determine the tissue distribution of *ASMT* genes.

## 2. Results

### 2.1. Copy Number Variation and Phylogenetic Relationships

All of the *ASMT* sequences were collected from 40 vertebrate species (Table 1 and Table S1). Previous studies reported that two *ASMT* genes are present in fish [18]. However, our present results confirmed that teleost genomes contain two or more *ASMT* genes. For example, many diploid teleosts possess two putative *ASMT* genes (*ASMT1* and *ASMT2*); however, the tetraploid *Sinocyclocheilus* fishes (Sg, Sr and Sa) possess two copies of the *ASMT2* gene (temporally named as *ASMT2a* and *ASMT2b*), although they displayed a high degree of conservation (over 93% similarity), reaching a total of three putative *ASMT* isotypes (Table 1). In contrast, only one *ASMT* gene was identified in the genomes of elephant shark (a cartilaginous fish) and Atlantic salmon. Moreover, our data demonstrated that teleost genomes possess only one *ASMTL* gene.

**Figure 1:**
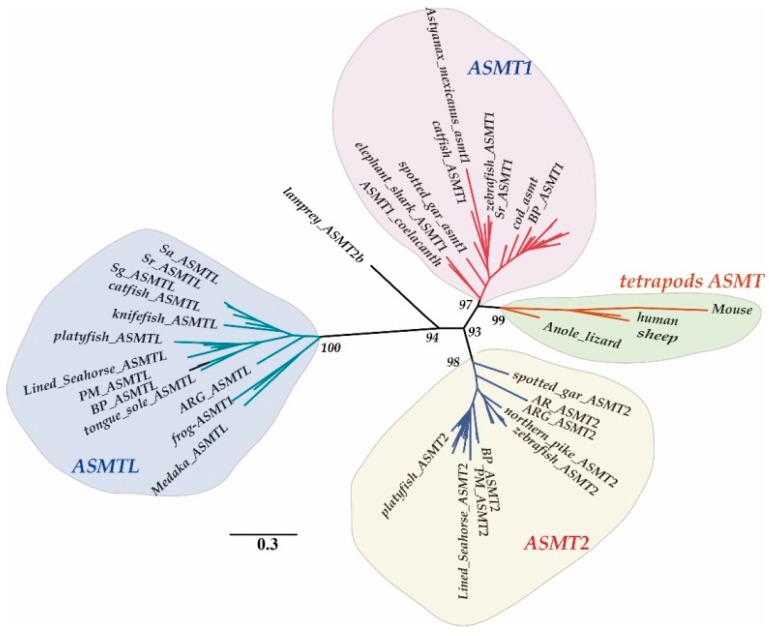
Clustering of *ASMT* genes. The phylogenetic analysis was performed by RAxML 8.0.17. Numbers on branches are bootstrap values. Scale bar indicates the rate of amino acid substitution per residue. Although the Mega tree is not shown here, it possesses the same tree topology. Different *ASMT* subfamilies are displayed with various colors: Pink, teleost *ASMT1*; Green, tetrapod *ASMT*; Yellow, teleost *ASMT2*; and Blue, teleost *ASMTL.* Abbreviations of fish species: PM, *Periophthalmus magnuspinnatus*; BP, *Boleophthalmus pectinirosris*; AR, Golden arowana; ARG, Green arowana; Sa, *Sinocyclocheilus anshuiensis;* Sg, *S. graham*; Sr, *S. rhinocerous.*

In addition, we performed a phylogenetic analysis using all the protein sequences of vertebrate ASMTs. On the basis of the constructed phylogenetic tree (Figure 1), we observed that fish *ASMT* genes form three distinct clades with high internal similarity (70.2–95.6%). The protein sequences of ASMTs were also aligned to our unpublished transcriptome database of Fish-T1K (Transcriptomes of 1000 fishes [23]; http://www.fisht1k.org/). Interestingly, we found that homologous sequences of these genes were present in almost all sequenced fish species (data not shown), suggesting a wide existence of the three *ASMT* genes in fishes.

### 2.2. Synteny Data

**Figure 2:**
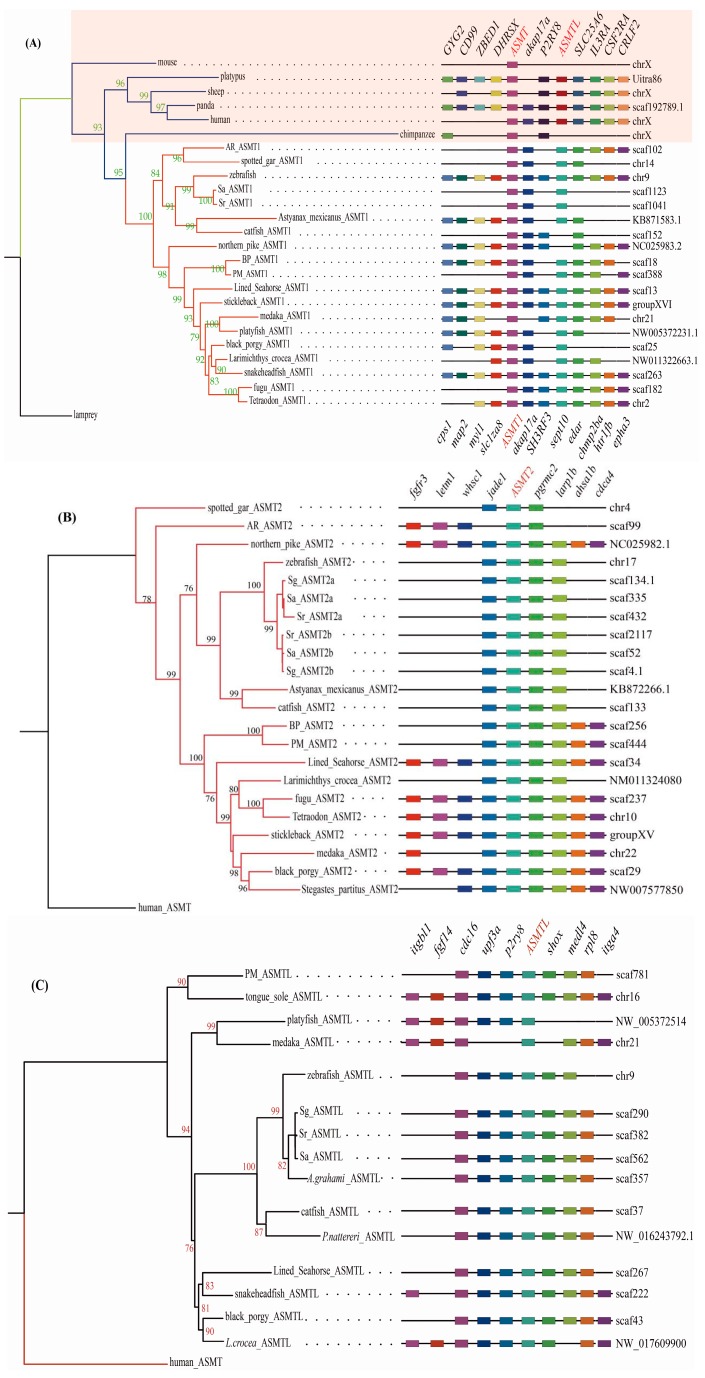
Phylogenetic trees and synteny of *ASMT1* (**A**), *ASMT2* (**B**), and *ASMTL* (**C**)*.* These ML trees (on the left side of each figure) were constructed by FastTree v2.1.7. Bootstrap values are shown on branches. The right figures are synteny of the three ASMT genes.

Our synteny analysis showed that *ASMT1* genes, and *ASMT2* genes, across species share a conserved suite of genes bounding them on both sides, although some species may display gene loss (Figure 2). In general, 11 genes (*GYG2*, *CD99*, *ZBED1*, *DHRSX*, *akap17a*, *P2RY8*, *ASMTL*, *SLC25A6*, *IL3RA*, *CSF2RA,* and *CRLF2*) usually flank the mammalian *ASMT* on both sides (the shadowed area in Figure 2A). Four (*cps1*, *map2*, *myl1,* and *slc1za8*) and seven genes (*akap17a*, *SH3RF3*, *sept10*, *edar*, *chmp2ba*, *htr1fb,* and *epha3*) reside on the upstream and the downstream regions respectively of fish *ASMT1* (Figure 2A). Four genes (*Fgfr3*, *letm1*, *whsc1,* and *jade1*) are located the upstream and four genes (*pgrmc2*, *larp1b*, *ahsa1b,* and *cdca4*) are positioned the downstream of fish *ASMT2* (Figure 2B). Moreover, nine genes reside around fish *ASMTL*, in which five genes (*itgbl1*, *fgf14*, *cdc16*, *upf3a,* and *p2ry8)* and four genes *(shox*, *medl4*, *rpl8,* and *itga4)* are located on the upstream and the downstream of fish *ASMTL*, respectively (Figure 2C).

Interestingly, we observed that *ASMT1* and *ASMTL* are sometimes located on the same chromosome or scaffold in vertebrates including fishes. For example, *ASMT* and *ASMTL* in humans reside on chromosome X, in zebrafish on chromosome 9, and in medaka on chromosome 21 (Figure 2A,C).

### 2.3. Structural Analysis of ASMT1, ASMT2 and ASMTL

**Figure 3:**
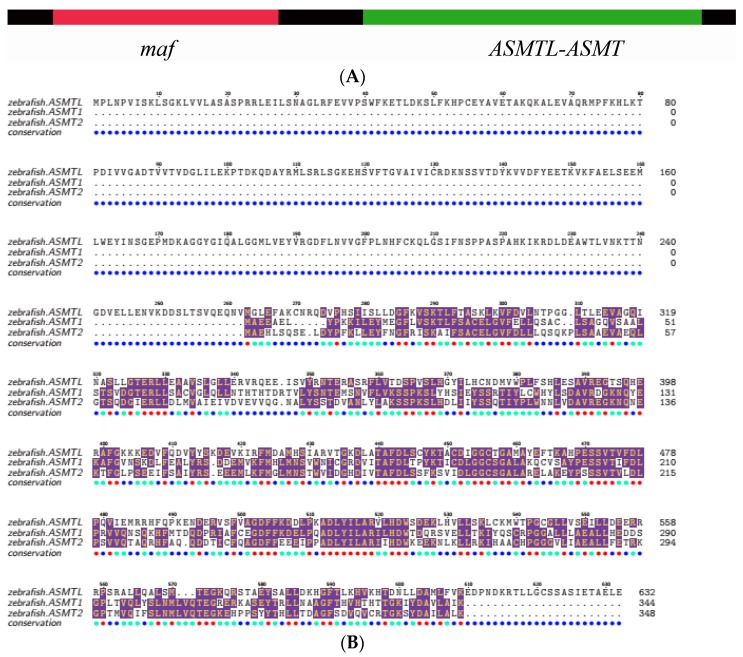
Alignment of ASMT protein sequences of zebrafish. (**A**) *ASMTL* has two different domains, with homology to two different genes (*maf* and *ASMTL-ASMT*; modified from [22]). (**B**) Similarity of the C-terminal of ASMTL (ASMTL-ASMT) to ASMT1 and ASMT2. See more details about the exon boundary in Figure S1.

**Figure 4:**
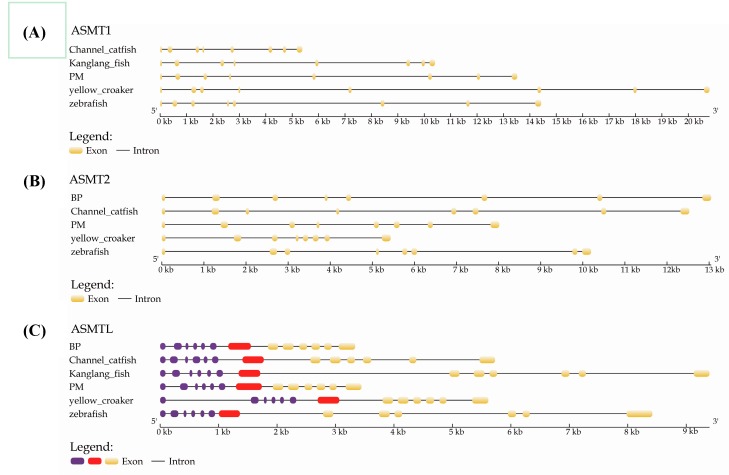
Exon-intron structures of *ASMT1* (**A**), *ASMT2* (**B**) and *ASMTL* (**C**)*.* Boxes in purple, red and yellow stand for the exons, while gray lines represent the introns. Note that the yellow boxes in (**C**) *ASMTL* are homologous to *ASMT1* and *ASMT2*.

A previous study [22] reported that *ASMTL* has two different domains, with close homology to two different genes (multicopy associated filamentation (*maf*) and *ASMT*; Figure 3A), with the C-terminal (ASMTL-ASMT) region homologous to the full length of the putative ASMTs (Figure 3B). We obtained the gene structures of *ASMT* members by comparing the zebrafish coding sequences and the collected genomic DNA sequences. Obviously, *ASMT1* and *ASMT2* are, respectively, conserved, sharing an independently similar pattern of exon-intron structure (Figure 4A,B), while they are remarkably different from the *ASMTL* style (Figure 4C). Meanwhile, all of the exon-intron boundaries are conserved. For example, BP-*ASMTL* is composed of 13 exons, and exons 8–13 (*ASMTL-ASMT*) are homologous to the *ASMT1* and *ASMT2* sequences (Figure S1 and Figure 3B). The Exon 7 (Figure S1) is the joining fragment between *maf* and *ASMTL-ASMT* (Figure 3A)*.*

### 2.4. The Structure of ASMT Proteins

**Figure 5:**
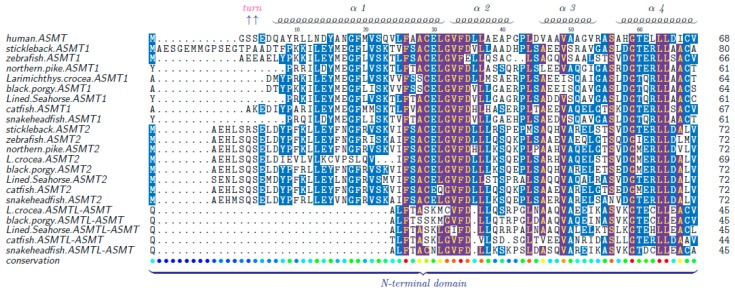

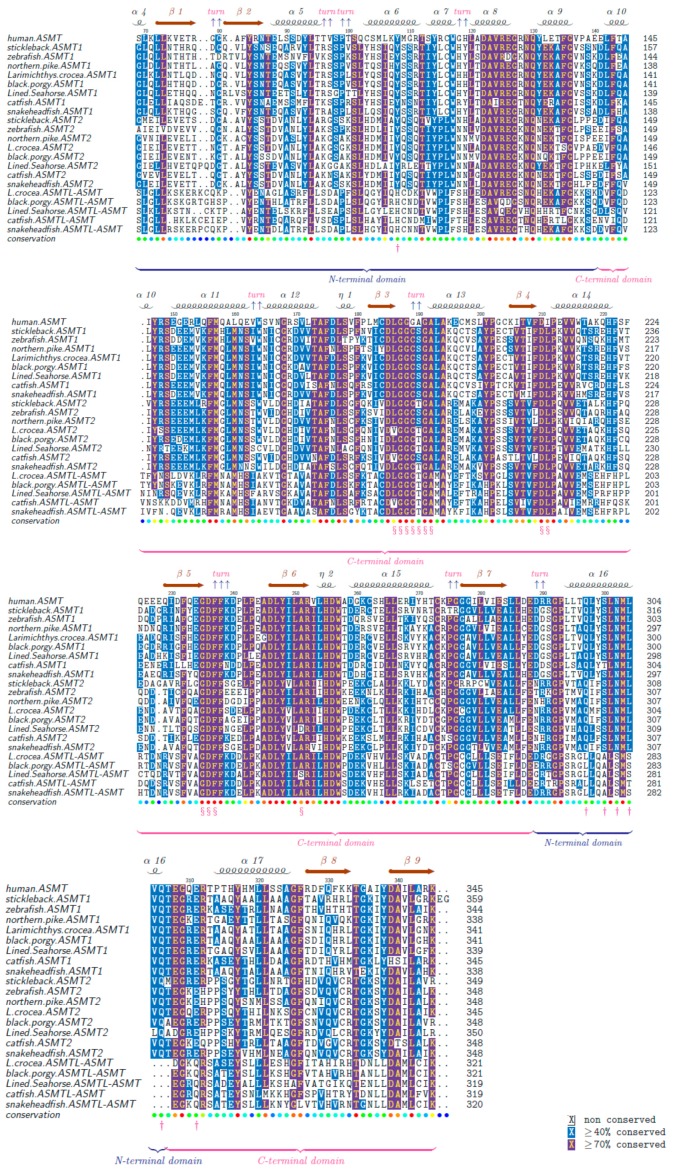
Alignment of ASMT protein sequences. These sequences were aligned with human ASMT by MAFFT and colorized using TEXshade. The secondary structural elements, alpha helix (α) and beta strand (β), are marked. Please note the remarkably conserved SAM binding residues (§). Residue differentiation between ASMTL-ASMT and other two ASMTs are marked using a dagger (†). Numbering is referred to the human ASMT. The color code for the conservation track ranges from red (the most conserved) to blue (the least conserved) as per TEXshade.

Representative ASMT protein sequences were aligned, and many residues were found to be well conserved (Figure 5). Previous studies reported that the polypeptide chain of ASMT comprises a C-terminal domain (a typical feature different from other SAM-dependent O-methyltransferases) and an *N*-terminal domain (intertwining several helices with another monomer to form the physiologically active dimer) [14]. Many residues around the SAM (S-adenosyl methionine) binding site are strictly conserved, such as the residues F156, G187, G235, D236, F237, F238, W257, D210, and R252. They may be helpful to maintain the H-bonds to the SAM moiety. At the NAS binding site, some residues are also well conserved between the sequences of ASMT1 and ASMT2, including Y108, F156, H255, D256, N302, M303, Q306, E311, and Y338. However, for these residues, some variants were present in the ASMTL-ASMT sequences. For example, Y108H, N302S and E311Q are among the obvious differences. Interestingly, previous findings revealed that some other variants can lead to reduced ASMT activity, such as E61Q and P243L [14], which were also identified in our current work (Figure 5).

### 2.5. Transcription and Cloning of ASMTs

Transcriptome data were analyzed to localize *ASMT* transcripts in different tissues of three representative *Sinocyclocheilus* fishes (Sg, *Sinocyclocheilus graham*; Sr, *S. rhinocerous;* and, Sa, *S. anshuiensis*) and two representative mudskippers (BP, *Boleophthalmus pectinirosris*; PM, *Periophthalmus magnuspinnatus*)*.* RPKM (Reads Per Kilobase Transcriptome per Million mapped reads) values (Table 2) were used to quantify gene transcription levels.

In the *Sinocyclocheilus* fishes, transcripts of four *ASMT* genes (*ASMT1*, *ASMT2a*, *ASMT2b,* and *ASMTL*) were determined in the analyzed tissues (Table 2). Our results demonstrated that *ASMT1* was the one most highly transcribed in the eyes, with the transcription level decreasing in the following order: Sg > Sr > Sa. Its transcription pattern is consistent with the habitat conditions of these fishes, since Sg is surface-dwelling, Sr is semi-cave dwelling and Sa is cave-restricted [24]. However, *ASMTL* was widely distributed in the examined tissues (eye, skin, liver and gonad) of Sr and Sa, although with differential transcription levels; whereas no *ASMTL* mRNA was detectable in Sg. Transcription of *ASMT2a* was observed in these *Sinocyclocheilus* fishes while *ASMT2b* was only highly transcribed in Sa. Meanwhile, we compared the transcriptome data from multiple tissues (liver, muscle, skin, gill and brain) of two mudskippers (Table 3). We observed that *ASMT2* and *ASMTL* displayed a wide distribution in the tissues of BP and PM, whereas *ASMT1* mRNA was only detectable in the brain (including the pineal gland) and the liver, with lower expression in the liver as compared to the brain.

Molecular cloning confirmed the cDNA sequences for *ASMT1*, *ASMT2,* and *ASMTL* genes derived from the genome of BP (corresponding accession numbers: MF787797, MF285271, and MF787798). As expected, BP-*ASMT1*, BP-*ASMT2,* and BP-*ASMTL* encode proteins with 344, 347 and 605 residues respectively.

### 2.6. Predicted Three-Dimensional (3D) Structures of Fish ASMTs

**Figure 6:**
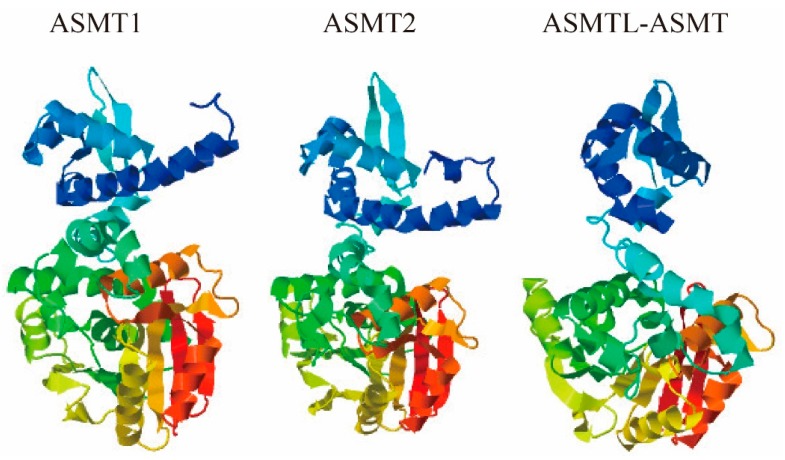
Predicted 3D models of ASMT1, ASMT2 and ASMTL-ASMT from BP.

The I-TASSER Suite was employed to predict 3D structures and potential functions of fish ASMT proteins. Three 3D atomic models with high accuracy for ASMT1, ASMT2 and ASMTL-ASMT in BP were generated (Figure 6), and their corresponding C-score values were calculated to be 1.58, 1.56, and 1.28, respectively. Similar structures suggested that all the three ASMT proteins may possess conserved ligand-binding sites to realize similar functions. We subsequently matched these predicted models to all available structures in the public PDB library, and interestingly, the best hit with the closest structure similarity occurred to the human ASMT protein (PDB ID: 4a6dA). The overall fold of the predicted fish ASMT dimers is very similar to the conditions in bacteria and plant *O*-methyltransferases (*O*-MTs), such as isoflavone *O*-MT and chalcone *O*-MT from alfalfa and isoflavone *O*-MT from barrel medic [14], which are SAM-dependent methyltransferases associated with secondary metabolism in alfalfa [25]. However, previous reports [14,26] suggested that the ASMT structures are in a “closed” configuration (similar to Figure 6), while chalcone *O*-MT from alfalfa and isoflavone *O*-MT from barrel medic are in an “open” construction.

## 3. Discussion

In this paper, we investigated many aspects of the fish *ASMT* family, and provide new information relating to the diversity, structural difference and tissue distribution of *ASMT*s in fish, from an integrated view of genomic and transcriptomic levels. The phylogenetic analysis, along with the synteny comparison, amino acid sequence alignment and protein structure prediction, revealed the presence of two putative *ASMT* isotypes (*ASMT1* and *ASMT2*) and a new *ASMTL* in teleost genomes. Meanwhile, we cloned the full-length cDNAs of *ASMT1*, *ASMT2* and *ASMTL* in the representative mudskipper BP.

Previous studies confirmed that all teleosts have gone through at least three rounds (Rs) of whole-genome duplication (WGD), with 1R and 2R before divergence of ray-fined fishes from jawed vertebrates [27,28,29]; some fish families even have undergone a fourth WGD [30,31]. According to our present results, we propose that variations in the number of *ASMT* genes in different fishes may be a result of WGD and gene loss, as in the case of arylalkylamine *N*-acetyltransferase (AANAT) [32,33], which is another important rate-limiting enzyme for melatonin biosynthesis. Tetrapods have only one *ASMT* gene, whereas the examined diploid fishes possess three isoforms and tetraploid teleosts like the *Sinocyclocheilus* fishes have even more copies of *ASMT* genes. The ratio of *ASMT* gene number between diploids and tetraploids is not always 1:2, due to possible gene loss as occurred in fish AANATs [32]. Moreover, the synteny regions of *ASMT1*, *ASMT2,* and *ASMTL* were well conserved across species, respectively. Interestingly, our data demonstrated that *ASMT1,* and *ASMTL* are located on the same chromosome, especially in mammals *ASMT1* and *ASMTL* are very close in localization. The *ASMT1* and *ASMT2* shared a conserved exon-intron pattern and the exons 8–13 of *ASMTL* are homologous to *ASMT1* and *ASMT2*.

In general, the WGD duplicates can escape the fate of gene loss when they obtain subfunctionalization or neofunctionalization [34,35]. For *ASMT*s, both putative isotypes (*ASMT1* and *ASMT2*) are present in most fishes, indicating that some subfunctionalization or neofunctionalization has occurred in these species. All of the ASMT enzymes and ASMTL-ASMT displayed strict conservation around the SAM-binding site, while differences were found at the NAS-binding site (no more than 73% identity). These differences may influence the stability and substrate selectivity of these enzymes. Altogether, these results suggest that ASMT1, ASMT2 and ASMTL may play differential functions in teleosts.

For a better understanding of the respective roles of *ASMTs*, we investigated their tissue distribution in several representative fish species. Our data indicated that *ASMT2* and *ASMTL* showed a wide transcriptional distribution, while *ASMT1* transcript was only localized in the eyes and the brain (not dissected from the pineal gland in our work), which are the main sources of melatonin biosynthesis. The wide presence of *ASMT2* transcript in fish peripheral organs is consistent with previous findings in goldfish [15,36,37], which proposed an earlier existence of the melatonin synthesizing system in peripheral tissues. A previous study also suggested a ubiquitous expression of *ASMTL* [22], which is similar to our present report. Conversely, the transcription of *ASMT1* was much more limited. As presented in our work, the eyes (including retinae) possess the highest transcription level of *ASMT1* among the tissues investigated in the three *Sinocyclocheilus* species, and only the brain (including the pineal gland) contained abundant *ASMT1* transcripts in the two mudskipper species. In agreement with a previous study in sea bass [10], our results may support the idea that *ASMT1* is mainly present in retinae and the pineal gland in fish for melatonin synthesis. It is interesting to note that the level of *ASMT1* transcription distinctly decreased in the following order: Sg > Sr > Sa, which is possibly associated with the regressive features of cave-restricted fish (such as eye degeneration and lack of circadian rhythms in Sa) [24]. In contrast, the transcription levels of *ASMTL* and *ASMT2b* were very high in the examined tissues of Sa as compared to those in Sg and Sr, suggesting that they may contribute to the compensatory evolution [24] and ASMTL may have a substrate other than NAS.

Generally speaking, protein structure determines protein’s function [38]. In order to support the possible function differences among the two putative ASMT isotypes and ASMTL-ASMT of ASMTL, we aligned their protein sequences and predicted related 3D structures (Figure 5 and Figure 6). We observed that these residues around the SAM binding sites are highly conserved among teleost ASMT1 and ASMT2, with only a few variations being noticed between them (such as N17E, I208V and R291S). Previous studies reported that these kinds of variations might play a strong impact on the ASMT activity [14]. A previous finding also suggested that ASMTL may be not associated with the melatonin pathway. Although the exact function of ASMTL is unclear, remarkable conservation of the putative catalytic domain for SAM binding support a methyltransferase activity of this enzyme. The notable difference distinguishing ASMTL-ASMT from ASMT1 and ASMT2 was located at the NAS binding sites, and some variations occurred (such as Y108H, N302S, E311Q), suggesting that ASMTL may have new function(s) in term of substrate selectivity.

## 4. Materials and Methods

### 4.1. Acquisition of ASMT and ASMTL for Nucleotide and Protein Sequences

A total of 40 vertebrate species, including 33 fish species were examined for our present research. These data were obtained by two ways. First, published ASMT and ASMTL sequences were downloaded from the public databases GenBank and Ensembl (Table S1). Second, unreported ASMT and ASMTL sequences from 21 fish species were derived from whole genome data generated by us and our collaborators. In detail, potential homology-based *ASMT* genes were retrieved from fish genomes using tBLASTn [39] with an e-value of 10^−5^. The BLAST results were subsequently processed by a perl script to obtain the best hit of each alignment. Finally, GeneWise v2.2.0 [40] was employed to predict the *ASMT* and *ASMTL* genes from the best hits.

### 4.2. Sequence Alignment and Phylogenetic Analysis

Nucleotide and protein sequences of all of the collected *ASMT* and *ASMTL* genes were used for further phylogenetic analysis. In brief, MAFFT software [41] was employed to align protein sequences of ASMTs and a Maximum Likelihood (ML) phylogenetic analysis was performed using RAxML 8.0.17 [42,43]. Additionally, ML phylogenetic trees of the three *ASMT* isotypes were constructed using their corresponding coding sequences by FastTree v2.1.7 [44]. We also downloaded from the public Protein Data Bank (PDB), a protein model of human ASMT (PDB code: 4A6D) for comparing structural differences among the fish ASMTs.

### 4.3. Analyses of Conserved Synteny and Gene Structures

For evaluating the conservation of *ASMT* genes, we checked several genes residing in the upstream and the downstream regions of each *ASMT* paralog. Related genomic data were obtained from GenBank and our lab as mentioned above. The stickleback (*Gasterosteus aculeatus*) genome [45] was used as the reference standard for searching any *ASMT* upstream and downstream regions. The genome assemblies of different fish species were searched using BLAST software, and the best hit was selected using a Perl script. GeneWise v2.2.0 [40] was employed to predict the ASMT gene structures.

### 4.4. Molecular Cloning of Mudskipper ASMT1, ASMT2 and ASMTL Transcripts

Total RNA from the eyes of blue-spotted mudskipper (*Boleophthalmus pectinirostris*, BP) [46] was extracted with TRIzol reagent (Invitrogen, Carlsbad, CA, USA). It was subsequently reverse-transcribed using the M-MuLV First Strand cDNA Synthesis Kit (Sangon, Shanghai, China) in 25-μL reactions, which were carried out for 50 min at 42 °C, followed by 15 min at 72 °C to inactivate the reverse transcriptase. These synthesized cDNAs were amplified using Q5 High-Fidelity DNA Polymerase (New England Biolabs, Ipswich, MA, USA) with specific primer pairs (Table S2) on a ABI 9700 thermal cycler (Life Technologies, Carlsbad, CA, USA) under the following cycling conditions: initial denaturation at 94 °C for 5 min; then 35 cycles of 94 °C for 30 s, 55 °C for 30 s and 72 °C for 30 s; and, final extension at 72 °C for 10 min. The amplified products were resolved in a 1.5% agarose gel, purified using a SanPrep Column DNA Gel Extraction Kit (Sangon, Shanghai, China), and sub-cloned into a pGEM-T Easy vector (Promega, Madison, WI, USA). Several positive clones of *E. coli* DH5α cells were picked for subsequent purification and sequencing validation.

### 4.5. Acquisition of Transcriptomic Data and Quantification of ASMT Transcripts

To investigate the tissue distribution of the *ASMT* genes, we selected two representative fish groups, tetraploid *Sinocyclocheilus* species and diploid mudskippers, for transcriptome analysis. Related transcriptomic data of four tissues (eye, skin, liver, and ovary) from three *Sinocyclocheilus* species that were previously generated by our lab [24] and have been deposited at NCBI Sequence Read Archive (SRA; accession numbers: *S. graham* (Sg), SRS1179797 to SRS1179800; *S. rhinocerous* (Sr), SRS1179996 to SRS1179999; and *S. anshuiensis* (Sa), SRS1180000 to SRS1180003). In addition, we previously produced transcriptomic data of five tissues (gill, skin, liver, muscle, and brain) from two representative mudskippers, BP and PM [46]. The Cufflink program 2.1.1 [47] with the core parameters (-FDR 0.05, -geometric-norm TRUE, -compatible-hits-norm TRUE) was employed to calculate the RPKM values of each *ASMT* gene, which are comparative parameters to quantify the relative transcription levels.

### 4.6. Tertiary Structure and Functional Prediction of Each ASMT Protein

As described previously [48], I-TASSER was employed to predict the tertiary structure and function of each ASMT. The confidence of models is quantitatively measured by a C-score that is calculated based on the significance of threading template alignments and the convergence parameters of the structure assembly simulations. A C-score is typically in the range of [−5, 2], where a high C-score supports the corresponding model with a high confidence.

## 5. Conclusions

In summary, we provide some novel insights into the fish *ASMT* family by a combination of comparative genomic and transcriptomic studies. We confirmed the existence of *ASMT1*, *ASMT2* and *ASMTL* in fish, and reported variations in the number of genes and the tissue distribution between species. We demonstrated that the C-terminal part of ASMTL (the ASMTL-ASMT region) is homology to the full length of ASMT1, and ASMT2, which is consistent with a previous report that two genes (*maf* and *ASMT*) were joined to form the *ASMTL* by gene fusion and duplication events. Our results also offer solid evidence to support previous findings that *ASMT1* is preferentially expressed in fish retinae and pineal gland, while *ASMT2* and *ASMTL* were mainly expressed in the fish peripheral tissues (such as liver, gut, skin, and gonad).

## Figures and Tables

**Table 1:** Copy number of *ASMT1*, *ASMT2,* and *ASMTL* genes in sequenced fish genomes.

Common Name	Species Name	*ASMT1*	*ASMT2*	*ASMTL*
Golden-line fishes (Sa) ^1^	*Sinocyclocheilus anshuiensis*	1	2	1
(Sg) ^1^	*Sinocyclocheilus grahami*	1	2	1
(Sr) ^1^	*Sinocyclocheilus rhinocerous*	1	2	1
Large yellow croaker	*Larimichthys crocea*	1	1	1
Northern snakehead	*Channa argus*	1	1	1
Giant-fin mudskipper	*Periophthalmus magnuspinnatus*	1	1	1
Blue-spotted mudskipper	*Boleophthalmus pectinirosris*	1	1	1
Lined seahorse	*Hippocampus erectus*	1	1	1
Kanglang fish	*Anabarilius grahami*	1	1	1
Black porgy	*Acanthopagrus schlegelii*	1	1	1
Southern platyfish	*Xiphophorus maculatus*	1	1	1
Bicolor damselfish	*Stegastes partitus*	1	1	1
Red-bellied piranha	*Pygocentrus nattereri*	1	1	1
Channel catfish	*Ictalurus punetaus*	1	1	1
Green arowana	*Scleropages formosus*	1	1	1
Golden arowana	*Scleropages formosus*	1	1	-
Red arowana	*Scleropages formosus*	1	1	-
Longjaw grenadier anchovy	*Coilia macrognathos*	1	1	-
Northern pike	*Esox lucius*	1	1	-
Atlantic salmon	*Salmo salar*	1	-	-
Elephant shark	*Callorhinchus milii*	1	-	-

-: unidentified. ^1^ Tetraploid fishes.

**Table 2:** Reads Per Kilobase Transcriptome per Million (RPKMs) of *ASMT* transcripts in three *Sinocyclocheilus* fishes.

Tissue	Gene	Sg	Sr	Sa
Eye	*ASMT1*	30.57	13.50	1.62
	*ASMT2a*	18.14	3.15	0.90
	*ASMT2b*	0.00	0.00	8.57
	*ASMTL*	0.00	3.35	122.11
Skin	*ASMT1*	0.09	0.07	0.19
	*ASMT2a*	7.36	2.71	2.07
	*ASMT2b*	0.00	0.35	5.02
	*ASMTL*	0.00	4.55	37.71
Liver	*ASMT1*	0.00	0.00	0.00
	*ASMT2a*	1.30	0.96	1.59
	*ASMT2b*	0.00	0.11	5.46
	*ASMTL*	0.00	71.22	118.35
Gonad	*ASMT1*	0.00	0.00	0.42
	*ASMT2a*	0.00	0.48	0.21
	*ASMT2b*	0.00	0.05	34.99
	*ASMTL*	0.00	17.71	14.78

**Table 3:** RPKMs of *ASMT* transcripts in two mudskippers.

Tissue	Gene	PM	BP
Brain	*ASMT1*	1.31	0.98
	*ASMT2*	8.47	2.43
	*ASMTL*	2.05	4.51
Gill	*ASMT1*	0.00	0.00
	*ASMT2*	2.43	2.43
	*ASMTL*	2.76	1.54
Liver	*ASMT1*	0.00	0.37
	*ASMT2*	3.09	1.73
	*ASMTL*	7.15	1.63
Muscle	*ASMT1*	0.00	0.00
	*ASMT2*	5.72	1.07
	*ASMTL*	3.01	1.64
Skin	*ASMT1*	0.00	0.00
	*ASMT2*	6.47	2.37
	*ASMTL*	4.93	0.85

## Supplementary Materials

The following are available online. Figure S1. Alignment of *ASMT* exon sequences from zebrafish. Table S1. The accession numbers for *ASMT* sequences. Table S2. Primer sequences used for molecular cloning of *ASMT*s and *ASMTL*.
