# Supplementary material for: A Comparative Genomic and Transcriptomic Survey Provides Novel Insights into N-Acetylserotonin Methyltransferase (ASMT) in Fish
Source: Molecules. 2017 Oct 2;22(10):1653. doi: 10.3390/molecules22101653 (PMC6151645; doi:10.3390/molecules22101653)
Supplement: Supplementary file 1 [file molecules-22-01653-s001.zip › supplementary materials/Figure S1.pdf]

exons 1-6

```
                            10        20        30        40        50        60        70        80
zebrafish_ASMTL   ATGCCGTTAAATCCAGTGATATCCAAGCTAAGTGGAAAGCTGGTTGTTCTGGCAAGCGCATCTCCACGACGCCTGGAGAT   80
zebrafish_ASMT1   ................................................................................    0
zebrafish_ASMT2   ................................................................................    0
consensus         ●●●●●●●●●●●●●●●●●●●●●●●●●●●●●●●●●●●●●●●●●●●●●●●●●●●●●●●●●●●●●●●●●●●●●●●●●●●●●●●●
```

homology to Maf

exons 1-6

```
                            90       100       110       120       130       140       150       160
zebrafish_ASMTL   TTTATCAAATGCTGGTTTACGATTTGAAGTTGTTCCCTCCTGGTTTAAAGAAACACTGGACAAGTCCTTGTTTAAGCACC  160
zebrafish_ASMT1   ................................................................................    0
zebrafish_ASMT2   ................................................................................    0
consensus         ●●●●●●●●●●●●●●●●●●●●●●●●●●●●●●●●●●●●●●●●●●●●●●●●●●●●●●●●●●●●●●●●●●●●●●●●●●●●●●●●
```

homology to Maf

exons 1-6

```
                           170       180       190       200       210       220       230       240
zebrafish_ASMTL   CATGTGAATATGCAGTGGAGACAGCTAAACAGAAGGCTTTGGAGGTGGCTCAACGAATGCCATTTAAACACTTGAAAACT  240
zebrafish_ASMT1   ................................................................................    0
zebrafish_ASMT2   ................................................................................    0
consensus         ●●●●●●●●●●●●●●●●●●●●●●●●●●●●●●●●●●●●●●●●●●●●●●●●●●●●●●●●●●●●●●●●●●●●●●●●●●●●●●●●
```

homology to Maf

exons 1-6

```
                           250       260       270       280       290       300       310       320
zebrafish_ASMTL   CCAGATATTGTTATCGGAGCGGACACTGTTGTGACTGTTGATGGCTTGATCTTGGAGAAGCCTACAGATAAACAAGATGC  320
zebrafish_ASMT1   ................................................................................    0
zebrafish_ASMT2   ................................................................................    0
consensus         ●●●●●●●●●●●●●●●●●●●●●●●●●●●●●●●●●●●●●●●●●●●●●●●●●●●●●●●●●●●●●●●●●●●●●●●●●●●●●●●●
```

homology to Maf

exons 1-6

```
                           330       340       350       360       370       380       390       400
zebrafish_ASMTL   TTACCGTATGCTGTCCAGGTTGAGCGGGAAGGAACATAGTGTCTTTACAGGTGTAGCGATCGTGATCTGCCGTGACAAAA  400
zebrafish_ASMT1   ................................................................................    0
zebrafish_ASMT2   ................................................................................    0
consensus         ●●●●●●●●●●●●●●●●●●●●●●●●●●●●●●●●●●●●●●●●●●●●●●●●●●●●●●●●●●●●●●●●●●●●●●●●●●●●●●●●
```

homology to Maf

exons 1-6

```
                           410       420       430       440       450       460       470       480
zebrafish_ASMTL   ATAGTTCAGTAACAGATTACAAAGTGGTTGATTTTTACGAAGAGACAAAAGTGAAGTTTGCAGAATTATCTGAGGAGATG  480
zebrafish_ASMT1   ................................................................................    0
zebrafish_ASMT2   ................................................................................    0
consensus         ●●●●●●●●●●●●●●●●●●●●●●●●●●●●●●●●●●●●●●●●●●●●●●●●●●●●●●●●●●●●●●●●●●●●●●●●●●●●●●●●
```

homology to Maf

exons 1-6 *exon 7*

```
                           490       500       510       520       530       540       550       560
zebrafish_ASMTL   CTTTGGGAATATATCAACAGTGGAGAGCCCATGGACAAGGCCGGTGGTTATGGTATTCAAGCTTTGGGTGGCATGTTGGT  560
zebrafish_ASMT1   ................................................................................    0
zebrafish_ASMT2   ................................................................................    0
consensus         ●●●●●●●●●●●●●●●●●●●●●●●●●●●●●●●●●●●●●●●●●●●●●●●●●●●●●●●●●●●●●●●●●●●●●●●●●●●●●●●●
```

homology to Maf

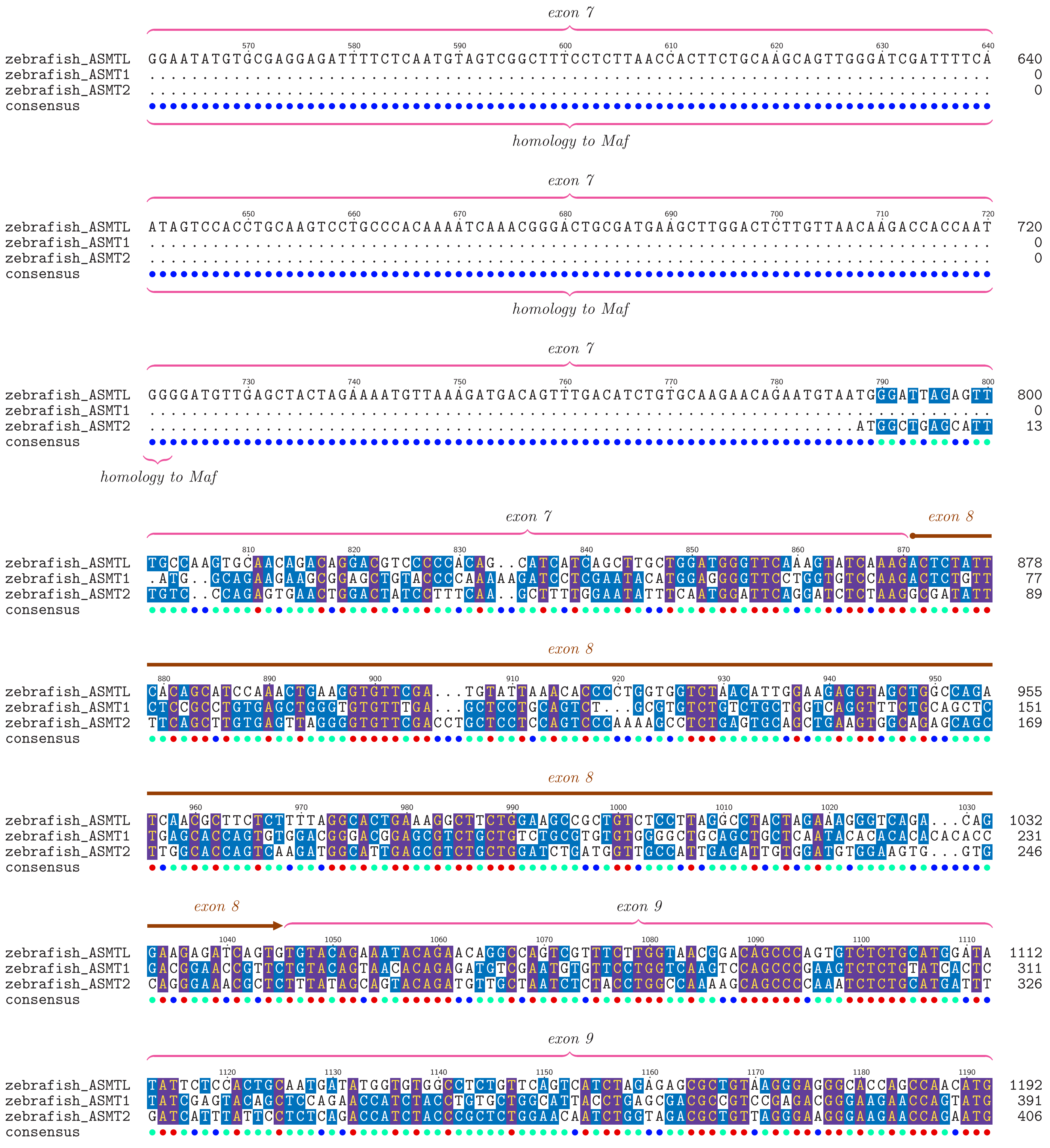
exon 7
homology to Maf
exon 8
exon 9
zebrafish_ASMTL
zebrafish_ASMT1
zebrafish_ASMT2
consensus
640 0 0
720 0 0
800 0 13
878 77 89
955 151 169
1032 231 246
1112 311 326
1192 391 406

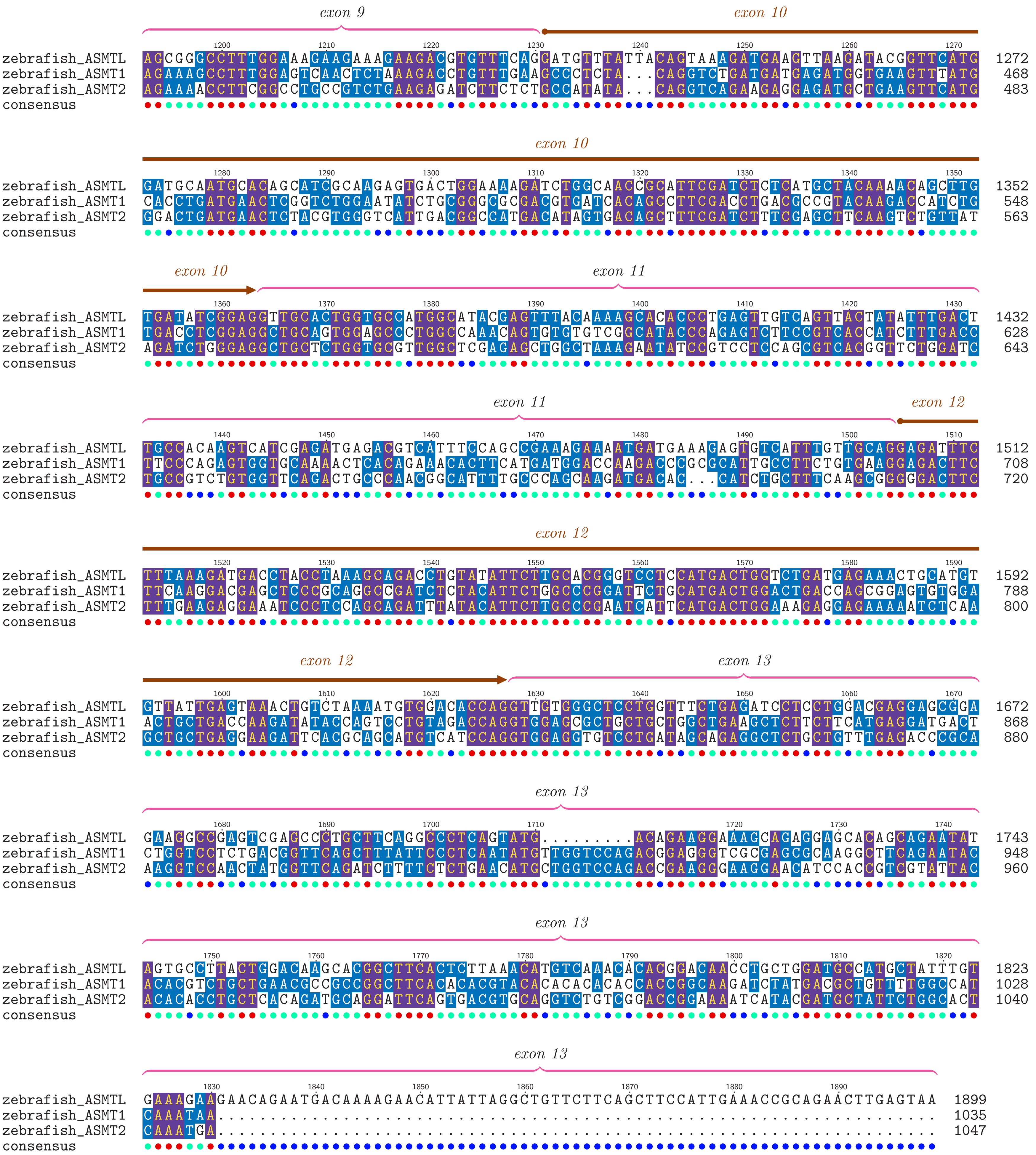
exon 9
exon 10
zebrafish_ASMTL 1272
zebrafish_ASMT1 468
zebrafish_ASMT2 483
consensus
exon 10
zebrafish_ASMTL 1352
zebrafish_ASMT1 548
zebrafish_ASMT2 563
consensus
exon 10
exon 11
zebrafish_ASMTL 1432
zebrafish_ASMT1 628
zebrafish_ASMT2 643
consensus
exon 11
exon 12
zebrafish_ASMTL 1512
zebrafish_ASMT1 708
zebrafish_ASMT2 720
consensus
exon 12
zebrafish_ASMTL 1592
zebrafish_ASMT1 788
zebrafish_ASMT2 800
consensus
exon 12
exon 13
zebrafish_ASMTL 1672
zebrafish_ASMT1 868
zebrafish_ASMT2 880
consensus
exon 13
zebrafish_ASMTL 1743
zebrafish_ASMT1 948
zebrafish_ASMT2 960
consensus
exon 13
zebrafish_ASMTL 1823
zebrafish_ASMT1 1028
zebrafish_ASMT2 1040
consensus
exon 13
zebrafish_ASMTL GAAAGAAGAACAGAATGACAAAAGAACATTATTAGGCTGTTCTTCAGCTTCCATTGAAACCGCAGAACTTGAGTAA 1899
zebrafish_ASMT1 CAAATAA 1035
zebrafish_ASMT2 CAAATGA 1047
consensus
