# Supplementary material for: A Comparative Genomic and Transcriptomic Survey Provides Novel Insights into N-Acetylserotonin Methyltransferase (ASMT) in Fish
Source: Molecules. 2017 Oct 2;22(10):1653. doi: 10.3390/molecules22101653 (PMC6151645; doi:10.3390/molecules22101653)
Supplement: Supplementary file 1 [file molecules-22-01653-s001.zip › supplementary materials/Table S2.docx]

**Table S2**. Primer sequences used for *ASMTs* and *ASMTL* cloning*.*

| Gene name | Primer name | Primer sequence ( from 5’ to 3’) |
| --- | --- | --- |
| BP_ASMT1 | BP_ASMT1-F1 | GCACAGGCTATAGAGGCGTACCC |
|  | BP_ASMT1-R1 | TTTGCGTCCCAGGACAGTGTCG |
| BP_ASMT2 | BP_ASMT2-F1 | ATGGCAGAACACCTTTCCCAAA |
|  | BP_ASMT2-R1 | TCTGATGGCGAGGATGGCGT |
| BP_ASMTL | BP_ASMTL-F1 | ATGGTACTGAACCCTGTCATTTC |
|  | BP_ASMTL-R1 | TTTGACACAGAGCATAGCGTCC |

F: forward primer; R: reverse primer
