# Supplementary material for: A Comparative Genomic and Transcriptomic Survey Provides Novel Insights into N-Acetylserotonin Methyltransferase (ASMT) in Fish
Source: Molecules. 2017 Oct 2;22(10):1653. doi: 10.3390/molecules22101653 (PMC6151645; doi:10.3390/molecules22101653)
Supplement: Supplementary file 1 [file molecules-22-01653-s001.zip › supplementary materials/Table S1.doc]

**Table S1. The accession numbers for ASMT sequences**

| **Class** | **Common Name** | **Species Name** | **Gene** | | **Nucleotide Sequence** | **Protein Sequence** | **Source** |
| --- | --- | --- | --- | --- | --- | --- | --- |
| Mammalia | human | *Homo sapiens* | | *ASMT* | NM_001171038.1 | P46597.1 | NCBI |
|  | chimpanzee | *Pan troglodytes* | | *ASMT* | ENSPTRT00000046289 | ENSPTRP00000042286 | Ensembl |
|  | Mouse | *Mus musculus* | | *ASMT* | NM_001199212.1 | NP_001186141.1 | NCBI |
|  | platypus | *Ornithorhynchus anatinus* | | *ASMT* | ENSOANT00000016668 | ENSOANP00000016665 | Ensembl |
|  | Sheep | *Ovis aries* | | *ASMT* | ENSOART00000008720 | ENSOARP00000008595 | Ensembl |
| Reptilia | Anole lizard | *Anolis carolinensis* | | *ASMT* | ENSACAT00000005929 | ENSACAP00000005801 | Ensembl |
|  | Turtle | *Pelodiscus sinensis* | | *ASMT* | ENSPSIT00000008477 | ENSPSIP00000008433 | Ensembl |
| Amphibia | Frog | *Xenopus laevis* | | *ASMT* | ENSXETT00000033600 | ENSXETP00000033600 | Ensembl |
| Actinopterygii | Medaka | *Oryzias latipes* | | *ASMT1* | ENSORLT00000019959 | ENSORLP00000019958 | Ensembl |
|  |  |  | | *ASMT2* | ENSORLT00000022485 | ENSORLP00000022484 | Ensembl |
|  |  |  | | *ASMTL* | XM_020712996.1 | XP_020568655.1 | NCBI |
|  | zebrafish | *Danio rerio* | | *ASMT1* | NM_001114909.1 | NP_001108381.1 | NCBI |
|  |  |  | | *ASMT2* | NM_001110477.1 | NP_001103947.1 | NCBI |
|  |  |  | | *ASMTL* | NM_213511.1 | NP_998676.1 | NCBI |
|  | Nile tilapia | *Oreochromis niloticus* | | *ASMT1* | ENSONIT00000005346 | ENSONIP00000005342 | Ensembl |
|  |  |  | | *ASMT2* | ENSONIT00000015611 | ENSONIP00000015598 | Ensembl |
|  | Spotted green pufferfish | *Tetraodon nigroviridis* | | *ASMT1* | ENSTNIT00000019757 | ENSTNIP00000019527 | Ensembl |
|  |  |  | | *ASMT2* | ENSTNIT00000015574 | ENSTNIP00000015369 | Ensembl |
|  | torafugu | *Takifugu rubripes* | | *ASMT1* | ENSTRUT00000014354 | ENSTRUP00000014288 | Ensembl |
|  |  |  | | *ASMT2* | ENSTRUT00000013120 | ENSTRUP00000013059 | Ensembl |
|  | Mexican tetra | *Astyanax mexicanus* | | *ASMT1* | ENSAMXT00000012991 | ENSAMXP00000012990 | Ensembl |
|  |  |  | | *ASMT2* | ENSAMXT00000018372 | ENSAMXP00000018372 | Ensembl |
|  | spotted gar | *Lepisosteus oculatus* | | *ASMT1* | ENSLOCT00000012584 | ENSLOCP00000012561 | Ensembl |
|  |  |  | | *ASMT2* | ENSLOCT00000013098 | ENSLOCP00000013070 | Ensembl |
|  | three-spined stickleback | *Gasterosteus aculeatus* | | *ASMT1* | ENSGACT00000008780 | ENSGACP00000008761 | Ensembl |
|  |  |  | | *ASMT2* | ENSGACT00000006073 | ENSGACP00000006056 | Ensembl |
|  | tongue sole | *Cynoglossus semilaevis* | | *ASMT* | XM_008328824.1 | XP_008327046 | NCBI |
|  |  |  | | *ASMTL* | XM_008328228.2 | XP_008326450.1 | NCBI |
|  | Atlantic cod | *Gadus morhua* | | *ASMT* | ENSGMOT00000000368 | ENSGMOP00000000347 | Ensembl |
| Chondrichthyes arcopterygii | Coelacanth | *Latimeria chalumnae* | | *ASMT* | ENSLACT00000016391 | ENSLACP00000016277 | Ensembl |
| Hyperoartia | sea lamprey | *Petromyzon marinus* | | *ASMT2a* | ENSPMAT00000002082 | ENSPMAP00000002072 | Ensembl |
